# Validation of the AI literacy questionnaire for Chinese pre-service teachers: psychometric evidence and profiles for differentiated educational evaluation

**DOI:** 10.3389/fpsyg.2026.1854432

**Published:** 2026-06-22

**Authors:** Jianpeng Liu, Jie An, Dongwei Liu, Shu Xu, Weiyi Zhang

**Affiliations:** 1 Guangzhou Huashang College, Guangzhou, China; 2 Research Base of Guangdong Basic Education Development, Guangzhou Huashang College, Guangzhou, China

**Keywords:** AI literacy, criterion-related validity, pre-service teachers, profile analysis, psychometric validation, teacher education

## Abstract

This study translated and adapted the AI Literacy Questionnaire for Chinese pre-service teachers, grounded in Expectancy-Value Theory as a pre-specified theoretical framework. Examining its psychometric properties in a sample of 341 pre-service teachers (78% female; 86.5% third-year students) from Guangdong Province, exploratory and confirmatory factor analyses supported a 24-item, five-factor structure: AI Ethics, AI Behavioral Commitment, AI Self-efficacy, AI Cognitive Application, and AI Intrinsic Motivation. The five-factor model showed acceptable fit and stable results across estimation methods, with the separation of self-efficacy and intrinsic motivation consistent with theoretical expectations. Internal consistency, convergent validity, and discriminant validity were supported. Configural invariance across gender was established, though metric and scalar invariance were not, likely attributable to the limited male sample size (*n* = 75). Criterion-related validity was partially supported, with AI Ethics, Behavioral Commitment, and Cognitive Application showing significant positive associations with AI teaching integration. Exploratory profile analysis identified four distinct profiles interpreted through Expectancy-Value Theory: Overall High-level Type (27.8%), Medium-level Type (41.3%), High Ethics-Low Self-efficacy Type (19.4%), and Overall Low-level Type (11.5%). The profiles showed significant grade development characteristics and differences in AI teaching integration ability, providing a basis for differentiated instructional approaches. Given sample characteristics, results should be interpreted with appropriate generalizability boundaries.

## 1 Introduction

The rapid development of Artificial Intelligence (AI) technology is profoundly transforming education worldwide. From intelligent tutoring systems to adaptive learning platforms, from automated assessment tools to generative AI applications such as ChatGPT and DeepSeek, AI technology is reshaping teaching and learning at all levels (Celik, 2023; Chiu and Chai, 2020; Chiu et al., 2021). The integration of AI-generated content into digital scenario-based teaching has demonstrated significant potential for enhancing pedagogical effectiveness in higher education contexts (Chen, 2007; Younas et al., 2025c), yet it also raises critical questions about educators’ preparedness to leverage these tools (Younas et al., 2025c). Recent systematic reviews of AI-based learning tools (2020–2025) reveal a rapidly evolving landscape where academic innovation depends fundamentally on educators’ capacity to understand, evaluate, and ethically deploy AI technologies (Younas et al., 2025a). UNESCO’s (2023) Global Report on AI in Education identifies AI literacy as one of the core competencies essential for citizens in the digital age (Kandlhofer et al., 2016; UNESCO, 2023). For educators, this competency is particularly critical as teachers serve not only as users of AI technologies but also as guides who shape students’ understanding and ethical use of these powerful tools. However, the relationship between AI technology and education is not uniformly positive: research has documented that perceived AI dependency can generate fear of missing out (FoMO) among students, potentially undermining the very learning outcomes that AI tools seek to enhance (Khoso et al., 2026). This tension underscores the need for educators who possess not only technical competence but also the ethical awareness and pedagogical judgment to harness AI’s potential while mitigating its risks. Consequently, teacher education programs bear significant responsibility for cultivating comprehensive AI literacy among pre-service teachers—those who are currently preparing to enter the teaching profession and will shape the next generation’s relationship with AI technology.

Despite the recognized importance of AI literacy (Long and Magerko, 2020), psychometrically validated instruments for assessing this construct remain limited, and significant debates persist regarding its conceptualization and measurement across educational contexts (Agarwal, 2019). Existing measures exhibit important limitations that constrain their applicability to teacher education. The 12-item AI Literacy Scale (Ng et al., 2021), while widely cited, reduces AI literacy to a unidimensional construct (Bloom, 1956), potentially obscuring the distinct motivational, behavioral, and ethical competencies that research has shown to differentially predict technology integration outcomes (Younas et al., 2025b). The MAILS scale (Carolus et al., 2023) offers a more comprehensive assessment but was developed primarily with Western adult populations and lacks validation for pre-service teachers in collectivist educational contexts where ethical responsibility and professional commitment carry distinct cultural meanings (Coşgun Ögeyik, 2022). Wang et al.’s (2023) instrument, though developed for non-computer science undergraduates, does not address the pedagogical application dimension essential for teacher readiness. The AI Literacy Questionnaire (AILQ) developed by Ng et al. (2024), grounded in the ABCE (Affective-Behavioral-Cognitive-Ethical) learning framework, addresses several of these limitations by capturing the multi-dimensional nature of AI literacy through 32 items across affective (intrinsic motivation, self-efficacy, confidence), behavioral (behavioral commitment, collaboration), cognitive (knowledge understanding, application-evaluation-creation), and ethical dimensions. Its theoretical grounding in the ABCE framework, which evolved from van Harreveld et al.’s (2015) ABC model with the addition of an ethical dimension, aligns well with the dual demands placed on teachers as both AI users and ethical role models. However, the AILQ was validated exclusively among Hong Kong secondary school students, and its psychometric properties (Cronbach’s *α* = 0.84–0.93; CFI = 0.95, RMSEA = 0.05) may not generalize to mainland Chinese pre-service teachers who differ systematically in educational stage, professional orientation, and learning context.

The need for independent validation of the AILQ for mainland Chinese pre-service teachers is further underscored by three critical considerations that prior validation studies have not adequately addressed. First, research on digital transformation and technological self-efficacy in AI-era education reveals that self-efficacy beliefs are profoundly shaped by institutional support structures and policy environments, which differ substantially between Hong Kong and mainland Chinese educational systems (Younas et al., 2026). China’s teacher education system operates within a distinct policy context characterized by large-scale national initiatives such as the “Education Informatization 2.0 Action Plan,” which explicitly mandates enhanced AI competencies for educators. This policy environment creates unique motivational dynamics where pre-service teachers’ AI learning is oriented toward future professional application rather than general interest, a distinction with important implications for how ability beliefs and task value should be measured and interpreted. Second, the pre-service teacher population in mainland China differs systematically from Hong Kong secondary students in educational stage (university vs. secondary), group identity (future professionals vs. general students), and learning contexts (career-oriented teacher preparation vs. general education). Cross-cultural research on AI literacy suggests that these differences influence not only the level but also the structure of AI literacy, particularly regarding the relationship between ethical cognition and behavioral engagement. Third, the integration of AI into China’s national curriculum system places unique demands on teachers’ AI competencies that extend beyond those required of general students, making assessment tools validated specifically for this population essential for effective teacher education policy and practice.

This study addresses three interconnected gaps in the existing literature. First, despite growing recognition of AI literacy’s importance for educators, no psychometrically validated instrument currently exists for assessing AI literacy among mainland Chinese pre-service teachers. While cross-cultural adaptation of psychological instruments is common, direct transfer of measures without validation in the target population risks compromising both measurement accuracy and practical utility, particularly when the source and target populations differ in educational stage, cultural context, and professional orientation. Second, existing AI literacy research has not adequately examined how established motivational theories can inform the structural organization of AI literacy dimensions. Recent studies on ChatGPT and intrinsic motivation in higher education demonstrate that technology acceptance models significantly predict student engagement, yet these theoretical insights have not been systematically applied to AI literacy instrument development or validation (Afzaal et al., 2025). Similarly, comprehensive reviews of AI-driven approaches to self-directed learning reveal that intrinsic motivation and self-efficacy are critical but distinguishable predictors of learning outcomes, suggesting that AI literacy instruments should theoretically anticipate and empirically verify the separation of these constructs (Younas et al., 2025b). Third, prior validation studies have not explored how AI literacy profiles might inform differentiated instruction in teacher education. The identification of heterogeneous learner profiles through person-centered approaches offers practical insights for tailoring AI literacy training to pre-service teachers with distinct motivational and competency patterns, yet this approach remains underutilized in AI literacy research. By addressing these gaps, this study provides the first systematic examination of the AILQ’s factor structure, reliability, and validity in a mainland Chinese pre-service teacher sample, grounded in Expectancy-Value Theory as a pre-specified theoretical framework.

The primary objectives of this study are: (1) to translate and culturally adapt the AILQ for use with mainland Chinese pre-service teachers; (2) to examine the factor structure of the adapted instrument through exploratory and confirmatory factor analyses; (3) to establish reliability (internal consistency) and validity (convergent, discriminant, and criterion-related) evidence; (4) to test measurement invariance across gender groups; and (5) to conduct exploratory profile analysis as supplementary evidence for differentiated AI literacy cultivation. Grounded in Expectancy-Value Theory, we pre-specify the hypothesis that the affective learning dimension of the ABCE framework will differentiate into distinct ability beliefs (self-efficacy) and task value (intrinsic motivation) factors in this population, given the professional orientation of pre-service teachers’ AI learning. However, we adopt an exploratory approach to determine the optimal factor structure, allowing data to inform the final model rather than imposing it *a priori*. The study is cross-sectional and does not examine longitudinal development or causal relationships. Additionally, due to sample characteristics (predominantly third-year students from Guangdong Province, with 78% female participants), results should be interpreted with appropriate consideration of generalizability boundaries, particularly regarding gender differences and grade-level development patterns.

Expectancy-Value Theory (EVT; Eccles and Wigfield, 2002) provides the pre-specified theoretical framework for this study. EVT posits that achievement motivation is shaped by two core beliefs: ability beliefs (expectations for success) and task value (perceived importance, interest, and utility). In the context of AI literacy among pre-service teachers, we theorize that self-efficacy (Bandura, 1977) corresponds to ability beliefs—confidence in one’s capacity to understand and apply AI technologies—while intrinsic motivation (Deci and Ryan, 1985) corresponds to task value—the perceived importance and interest of AI learning for future teaching practice. Based on EVT, we derive three specific expectations for this validation study. First, we hypothesize that the affective learning dimension of the original ABCE framework will differentiate into separate self-efficacy and intrinsic motivation factors because pre-service teachers’ AI learning is oriented toward future professional application rather than general interest, making ability beliefs and task value more psychologically distinct than among general student populations. Second, we expect that these differentiated motivational dimensions will show differential predictive relationships with AI teaching integration ability, consistent with EVT’s proposition that both ability beliefs and task value contribute to achievement outcomes but through partially distinct pathways. Third, we anticipate that profile analysis will reveal distinct motivational patterns corresponding to different combinations of ability beliefs and task value (e.g., high task value with low ability beliefs), which have different implications for instructional intervention. It is important to note that while EVT guides our theoretical expectations, the factor structure itself is determined empirically through exploratory and confirmatory analyses, allowing the data to inform the final model while providing a coherent theoretical framework for interpreting results.

## 2 Method

### 2.1 Participants and procedure

Participants were recruited from eight universities in Guangdong Province through class-based survey administration. All participants were undergraduate students enrolled in teacher education programs. Data collection took place between March and May 2024. The survey was administered online via Wenjuanxing (a Chinese survey platform similar to Qualtrics) during regular class hours, with instructors providing time for students to complete the questionnaire. Participation was voluntary, and no compensation was provided.

Prior to data collection, the study protocol was reviewed and approved by the Institutional Research Ethics Committee [blinded for review]. Informed consent was obtained electronically at the beginning of the survey, with participants required to click “I agree to participate” before proceeding to the questionnaire items. Participants were informed that they could withdraw at any time without penalty and that their responses would be kept confidential.

The final sample comprised 341 participants (75 males, 266 females; Gender was coded as 0 = male, 1 = female.) after data screening. Gender was assessed using a single self-report item asking “What is your gender?” with response options of “Male,” “Female,” and “Other/Prefer not to say.” No participants selected “Other/Prefer not to say.” We use the term “gender” rather than “sex” because the construct of interest relates to social and psychological identity rather than biological characteristics, and because the survey item assessed self-identified gender. Grade distribution was: first year (*n* = 2, 0.6%), second year (*n* = 35, 10.3%), third year (*n* = 295, 86.5%), and fourth year (*n* = 9, 2.6%). All participants were from teacher education majors. The small subsample sizes for first-year (*n* = 2) and fourth-year (*n* = 9) precluded meaningful grade-based comparisons beyond the second-vs-third-year contrast; thus, any grade-related findings should be considered hypothesis-generating rather than confirmatory (see Table 1).

**Table 1:** Sample characteristics (*N* = 341).

Characteristic	Category	Raw sample	Valid sample
Gender	Male	82/20.7%	75/22.0%
	Female	315/79.3%	266/78.0%
Grade	First year	5/1.3%	2/0.6%
	Second year	42/10.6%	35/10.3%
	Third year	340/85.6%	295/86.5%
	Fourth year	10/2.5%	9/2.6%
Major	Teacher education	397/100.0%	341/100.0%

### 2.2 Instrument and translation

The AI Literacy Questionnaire (AILQ; Ng et al., 2024) was used as the source instrument. The original scale comprises 32 items measuring four dimensions: affective learning (10 items), behavioral learning (8 items), cognitive learning (6 items), and ethical learning (8 items). Items are rated on a 5-point Likert scale (1 = strongly disagree to 5 = strongly agree). The original scale demonstrated good reliability (Cronbach’s *α* = 0.84–0.93) and validity (CFI = 0.95, RMSEA = 0.05) in a Hong Kong secondary school sample.

The scale was translated following Brislin’s (1970, 1986) translation model: (1) two bilingual education doctoral students independently translated the English items into Chinese; (2) two English-major graduate students back-translated the Chinese version into English; (3) a panel of five experts in educational technology and psychology reviewed both versions for semantic accuracy, cultural appropriateness, and linguistic fluency; and (4) pilot testing with 30 pre-service teachers led to minor wording adjustments. The final Chinese version retained all 32 original items with culturally adapted expressions.

### 2.3 Criterion measure

AI teaching integration ability was assessed as the criterion variable to examine the validity of the AI literacy scores. This construct was measured using an adapted version of the Technological Pedagogical Content Knowledge (TPACK) framework specifically contextualized for AI education. The scale comprised 8 items assessing pre-service teachers’ perceived ability to integrate AI tools into their teaching practice, rated on a 5-point Likert scale (1 = strongly disagree to 5 = strongly agree). Sample items include “I can design lesson plans that effectively integrate AI tools with subject content” and “I can select appropriate AI tools to support student learning in my subject area.” In the current sample, the scale demonstrated good internal consistency (Cronbach’s *α* = 0.87, McDonald’s *ω* = 0.88).

This criterion was selected because AI literacy among pre-service teachers should ultimately translate into their ability to effectively integrate AI technologies into their future teaching practice. The TPACK framework provides a comprehensive theoretical foundation for understanding how teachers integrate technology, pedagogy, and content knowledge, making it particularly appropriate for assessing AI teaching integration ability in a teacher education context.

### 2.4 Data screening and statistical analysis

#### 2.4.1 Data screening

Careless responses were identified using multi-indicator screening: (1) longstring (≥15 consecutive identical responses), (2) low variability (SD < 0.3 across all items), and (3) restricted range (range ≤1). Participants meeting ≥2 criteria were excluded. Detailed screening results are provided in Supplementary file 1.

#### 2.4.2 Preliminary item analysis and EFA

Item distributions were examined to identify skewness and category usage patterns. Exploratory factor analysis (EFA) was conducted using MinRes extraction with Promax oblique rotation. The number of factors was determined through multiple criteria: Kaiser criterion (eigenvalue >1), scree plot inspection, parallel analysis, and theoretical interpretability. EFA was based on Pearson correlation matrices as an exploratory step; the ordinal nature of the data was primarily addressed through subsequent WLSMV confirmatory factor analysis.

#### 2.4.3 CFA and model comparison

Confirmatory factor analysis (CFA) was conducted using WLSMV (weighted least squares mean and variance adjusted) as the primary estimation method, with ML/MLR (maximum likelihood with robust standard errors) as a sensitivity check. Model fit was evaluated using CFI, TLI, RMSEA, and SRMR. Alternative models (four-factor, five-factor, six-factor, Bifactor, ESEM) were compared to determine the optimal structure.

#### 2.4.4 Additional analyses

Internal consistency reliability was assessed using Cronbach’s *α* and McDonald’s *ω*. Convergent validity was evaluated through AVE and CR, and discriminant validity through the HTMT criterion. Measurement invariance across gender groups was tested using multi-group CFA (configural, metric, scalar; Cheung and Rensvold, 2002; Meade and Craig, 2012). Criterion-related validity was examined through SEM path analysis predicting AI teaching integration. Exploratory K-means cluster analysis was conducted to identify potential AI literacy profiles, with the number of clusters determined by silhouette coefficient, Calinski-Harabasz index, and elbow method. All analyses were performed using R (psych, lavaan packages) and Python 3.12.

## 3 Results

### 3.1 Data quality and item characteristics

Raw data contained 397 samples; after multi-indicator joint screening, 56 problematic samples (14.1%) were excluded, yielding 341 valid samples for subsequent analysis. Missing value analysis showed that all 32 items had no missing values in the cleaned data (missing rate = 0.00%), indicating good data integrity.

Descriptive statistical analysis revealed that 96.9% of items (31 out of 32) exhibited significant skewness or unbalanced category usage, suggesting a high endorsement tendency across items. These distribution characteristics support treating the items as ordinal categorical variables and using WLSMV/DWLS estimation for subsequent CFA.

### 3.2 Factor retention and EFA results

Before conducting EFA, sampling adequacy was assessed. The Kaiser-Meyer-Olkin (KMO) measure was 0.91, and Bartlett’s test of sphericity was significant (*χ*^2^ (496) = 5551.24, *p* < 0.001), indicating data suitability for factor analysis.

Multiple criteria were used to determine the number of factors (Kline, 1994). Kaiser criterion (eigenvalue >1) suggested retaining six factors, whereas the scree plot and parallel analysis supported retaining five factors (the sixth eigenvalue of 1.06 fell below the 95th percentile of random data at 1.21). Considering factor interpretability and parsimony, a five-factor solution was retained for further testing.

**Figure 1:**
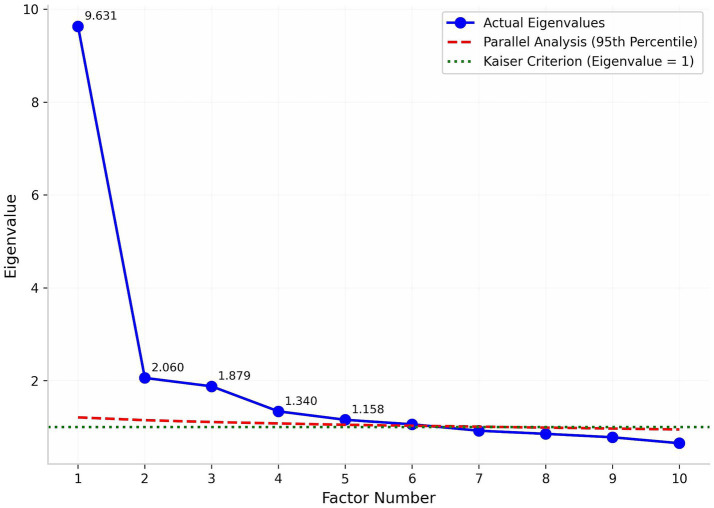
Scree plot and parallel analysis.

The five-factor structure accounted for 59.28% of total variance. After deleting 8 items with low loadings or serious cross-loadings, the final scale comprised 24 items: AI Ethics (8 items, 30.10% variance), AI Behavioral Commitment (4 items, 12.69% variance), AI Self-efficacy (4 items, 6.44% variance), AI Cognitive Application (4 items, 5.87% variance), and AI Intrinsic Motivation (4 items, 4.19% variance). The deletion of these items was guided by both statistical criteria and theoretical considerations. Four behavioral collaboration items failed to form an independent factor, consistent with the interpretation that pre-service teachers’ collaborative behaviors are embedded within specific professional task commitments rather than manifesting as independent behavioral characteristics. Two affective items cross-loaded between self-efficacy and intrinsic motivation, reflecting the theoretical proposition from Expectancy-Value Theory that ability beliefs and task value, while related, are distinguishable constructs—particularly in professional preparation contexts where confidence and interest have distinct antecedents. Two cognitive knowledge items exhibited low communalities, likely reflecting a ceiling effect given the integration of basic AI concepts into Guangdong’s pre-service teacher curriculum; the retained cognitive application items better capture the practical knowledge dimension relevant to teaching practice. Table 2 presents the factor structure summary, and Supplementary file 2 provides detailed theoretical justification for each deleted item (see Figure 1).

**Table 2:** Representative EFA loadings for the retained five-factor solution.

Item	Factor 1 (AI ethics)	Factor 2 (AI behavioral commitment)	Factor 3 (AI self-efficacy)	Factor 4 (AI cognitive application)	Factor 5 (AI intrinsic motivation)
DA12	0.855	—	—	—	—
DA10	0.833	—	—	—	—
DA5	0.821	—	—	—	—
BA3	—	0.860	—	—	—
BA2	—	0.812	—	—	—
AB2	—	—	0.870	—	—
AB4	—	—	0.823	—	—
CA5	—	—	—	0.852	—
CB4	—	—	—	0.793	—
AA3	—	—	—	—	0.899
AA1	—	—	—	—	0.806

### 3.3 CFA results and model comparison

Given the ordinal nature of the data, confirmatory factor analysis was conducted using WLSMV as the primary estimation method. The five-factor model demonstrated acceptable fit: CFI = 0.96, TLI = 0.95, RMSEA = 0.035, SRMR = 0.069. All standardized factor loadings exceeded 0.60 (range: 0.60–0.81), indicating strong item-factor relationships.

To verify cross-method robustness, ML/MLR estimation was conducted as a sensitivity check. Results showed consistent findings: CFI = 0.95, TLI = 0.94, RMSEA = 0.050, SRMR = 0.068. Differences between WLSMV and ML estimates were minimal (ΔCFI = 0.01, ΔRMSEA = 0.015), supporting the stability of the five-factor structure across estimation methods.

Alternative model comparisons were conducted to evaluate the relative appropriateness of different factor structures (Table 3). The five-factor model showed substantially better fit than the four-factor model (ΔCFI = 0.10, ΔRMSEA = 0.035). Compared to the six-factor model, the five-factor solution was more parsimonious while achieving comparable fit; the sixth factor was marginal (eigenvalue = 1.06) and lacked clear theoretical interpretation. Both ESEM and Bifactor models showed inferior fit to the five-factor solution. For educational evaluation, this five-factor structure provides a multidimensional framework for assessing AI literacy, allowing each dimension to be used independently for diagnostic assessment (e.g., identifying specific weaknesses in AI Self-efficacy or AI Ethics).

**Table 3:** Model comparison fit indices.

Index	4-factor model	5-factor model	6-factor model	ESEM model	Bifactor model	Criterion
*χ* ^2^	855.11	451.13	427.20	—	—	—
d*f*	246	242	237	—	—	—
*χ*^2^/d*f*	3.48	1.86	1.80	2.31	2.05	<3
CFI	0.841	0.945	0.950	0.897	0.921	>0.90
TLI	0.822	0.938	0.942	—	—	>0.90
RMSEA	0.085	0.050	0.049	0.062	0.057	<0.08
GFI	0.792	0.890	0.896	—	—	>0.90
AGFI	0.767	0.875	0.879	—	—	>0.80
NFI	—	0.890	—	—	—	>0.90
SRMR	—	0.068	0.065	—	—	≤0.08
AIC	—	112.85	—	—	—	Smaller better
BIC	—	335.10	—	—	—	Smaller better

For models estimated with WLSMV (the five-factor model), the *χ*^2^/d*f* ratio is reported for descriptive purposes only; model fit was primarily evaluated using CFI, TLI, RMSEA, and SRMR.

The superior fit of the five-factor model over the original four-factor structure suggests that for pre-service teachers, AI literacy should be assessed as a multidimensional construct with separate self-efficacy and intrinsic motivation components (see Table 4).

**Table 4:** Standardized factor loadings for the retained five-factor model.

Dimension	Item	WLSMV/DWLS loading	ML loading	Difference	SE (WLSMV)	*z*-value	Residual variance
AI ethics	DA2	0.599	0.612	0.013	0.045	13.38***	0.637
	DA5	0.751	0.768	0.017	0.038	19.76***	0.436
	DA7	0.739	0.754	0.015	0.039	18.95***	0.454
	DA8	0.654	0.671	0.017	0.043	15.21***	0.572
	DA10	0.788	0.802	0.014	0.036	21.89***	0.379
	DA11	0.746	0.761	0.015	0.038	19.63***	0.443
	DA12	0.787	0.801	0.014	0.036	21.86***	0.381
	DA13	0.745	0.759	0.014	0.038	19.61***	0.445
AI behavioral commitment	BA2	0.723	0.738	0.015	0.041	17.63***	0.477
	BA3	0.776	0.791	0.015	0.038	20.42***	0.398
	BA4	0.795	0.809	0.014	0.037	21.49***	0.368
	BA5	0.750	0.764	0.014	0.039	19.23***	0.438
AI self-efficacy	AB1	0.780	0.794	0.014	0.037	21.08***	0.392
	AB2	0.805	0.819	0.014	0.035	23.00***	0.352
	AB3	0.719	0.734	0.015	0.041	17.54***	0.483
	AB4	0.789	0.803	0.014	0.036	21.92***	0.377
AI cognitive application	CA1	0.643	0.659	0.016	0.044	14.61***	0.587
	CA5	0.713	0.728	0.015	0.041	17.39***	0.492
	CB3	0.695	0.711	0.016	0.042	16.55***	0.517
	CB4	0.797	0.812	0.015	0.037	21.54***	0.365
AI intrinsic motivation	AA1	0.709	0.724	0.015	0.041	17.29***	0.497
	AA2	0.778	0.792	0.014	0.038	20.47***	0.395
	AA3	0.778	0.793	0.015	0.038	20.47***	0.395
	AA4	0.716	0.731	0.015	0.041	17.46***	0.487

***p < 0.001.

### 3.4 Reliability and construct validity

Internal consistency was acceptable to strong across factors. Cronbach’s *α* ranged from 0.80 to 0.90 (Nunnally, 1978), and McDonald’s *ω* ranged from 0.81 to 0.90. Composite reliability (CR) exceeded 0.84 for all factors, and average variance extracted (AVE) ranged from 0.57 to 0.65, supporting convergent validity (Table 5).

**Table 5:** Reliability and convergent validity.

Dimension	Cronbach’s *α*	*ω* coefficient	AVE	AVE evaluation	CR
AI ethics	0.8979	0.903	0.571	Good	0.913
AI behavioral commitment	0.8461	0.850	0.644	Good	0.878
AI self-efficacy	0.8551	0.860	0.645	Good	0.879
AI cognitive application	0.8016	0.812	0.575	Good	0.843
AI intrinsic motivation	0.8312	0.830	0.605	Good	0.857

Discriminant validity was assessed using the HTMT criterion. All HTMT values were below 0.85 (range: 0.21–0.58), indicating adequate discriminant validity among the five factors (Table 6). Factor correlations ranged from 0.21 to 0.58, showing both theoretical associations and sufficient distinctiveness.

**Table 6:** Discriminant validity (HTMT).

Factor pair	HTMT value	Evaluation
AI ethics–AI behavioral commitment	0.388	Good
AI ethics–AI self-efficacy	0.246	Good
AI ethics–AI cognitive application	0.205	Good
AI ethics–AI intrinsic motivation	0.407	Good
AI behavioral commitment–AI self-efficacy	0.504	Good
AI behavioral commitment–AI cognitive application	0.580	Good
AI behavioral commitment–AI intrinsic motivation	0.492	Good
AI self-efficacy–AI cognitive application	0.451	Good
AI self-efficacy–AI intrinsic motivation	0.403	Good
AI cognitive application–AI intrinsic motivation	0.266	Good

### 3.5 Gender invariance

Multi-group confirmatory factor analysis was conducted to test measurement invariance across gender groups. Configural invariance across gender was supported (CFI = 0.94, RMSEA = 0.048), indicating that the same five-factor structure is conceptually meaningful for both male and female participants. This foundational level of invariance establishes that the five AI literacy dimensions are understood similarly across gender groups, which is the prerequisite for any meaningful cross-group comparison. However, metric invariance (ΔCFI = 0.014) and scalar invariance were not established, raising important considerations for score interpretation. The failure to achieve metric invariance suggests that some items may function differently across gender groups—that is, a given change in the latent construct may not correspond to the same expected change in item responses for male and female participants. Several factors may contribute to this finding. First, the limited male sample size (*n* = 75) falls below the recommended threshold of 100–200 for robust measurement invariance testing (Meade and Bauer, 2007), potentially compromising statistical power to detect invariant factor loadings. Second, genuine differences in how male and female pre-service teachers interpret specific item content may exist; for example, ethical items regarding AI fairness and privacy may carry different salience across gender groups in the Chinese cultural context. Third, the predominantly female composition (78%) reflects the broader demographic pattern in Chinese teacher education programs, but may limit the precision of parameter estimates for male participants. Given these limitations, we recommend that researchers and practitioners: (a) avoid comparing latent means or observed total scores across gender using this instrument until stronger invariance is established with more balanced samples; (b) consider item-level comparisons or alignment optimization approaches if gender comparisons are necessary; and (c) report gender-specific norms separately rather than pooling scores. Future research with more balanced gender samples should replicate these analyses and explore potential item bias through differential item functioning (DIF) analysis (see Table 7).

**Table 7:** Measurement invariance test across gender.

Invariance level	*χ* ^2^	d*f*	CFI	RMSEA	ΔCFI	ΔRMSEA	Invariance judgment
Configural	480.52	484	0.942	0.048	—	—	Supported
Metric	528.36	508	0.928	0.055	0.014	0.007	Not supported
Scalar	565.48	532	0.921	0.058	0.007	0.003	Not supported

### 3.6 Criterion-related validity

Structural equation modeling was conducted to examine the relationship between AI literacy factors and AI teaching integration ability. The SEM model showed acceptable fit: *χ*^2^ (284) = 836.72, *p* < 0.001; CFI = 0.90; TLI = 0.88; RMSEA = 0.066 (90% CI: 0.061–0.072).

Three factors showed significant positive associations with AI teaching integration: AI Ethics (*β* = 0.22, *p* = 0.041), AI Behavioral Commitment (*β* = 0.29, *p* < 0.001), and AI Cognitive Application (*β* = 0.13, *p* = 0.019). AI Self-efficacy was not significantly associated (*β* = 0.01, *p* = 0.89), and AI Intrinsic Motivation showed only marginal significance (*β* = 0.08, *p* = 0.091). The model explained 28% of variance in AI teaching integration ability (*R*^2^ = 0.28). From an evaluation perspective, the significant predictive effects of AI Ethics, Behavioral Commitment, and Cognitive Application (*β* = 0.13–0.29) suggest that these three dimensions should receive greater weight in summative assessments of pre-service teachers’ readiness to integrate AI into teaching. In contrast, AI Self-efficacy and Intrinsic Motivation, which showed non-significant or marginal effects, may be more suitable as targets for formative assessment rather than high-stakes evaluation.

### 3.7 Exploratory profile analysis

As supplementary exploration, K-means cluster analysis was conducted to identify potential profiles of pre-service teachers’ AI literacy. The four-cluster solution was retained based on silhouette coefficient (0.72), Calinski-Harabasz index (186.3), and elbow method, all indicating that four clusters represent the optimal solution with good statistical validity.

Four distinct profiles were identified (Table 8):

1. Overall high-level type (27.8%): This group demonstrates high scores across all five dimensions (AI Ethics *M* = 4.23, AI Self-efficacy *M* = 4.15, AI Intrinsic Motivation *M* = 4.08, AI Behavioral Commitment *M* = 4.02, AI Cognitive Application *M* = 3.97). These pre-service teachers exhibit strong AI ethical awareness, sufficient self-efficacy and intrinsic motivation, and actively engage in AI learning while proficiently applying relevant knowledge. They serve as the benchmark group for AI literacy cultivation.
2. Medium-level type (41.3%): As the largest group in the sample, these pre-service teachers show relatively balanced development across all dimensions but at medium levels (AI Ethics *M* = 3.65, AI Self-efficacy *M* = 3.58, AI Intrinsic Motivation *M* = 3.52, AI Behavioral Commitment *M* = 3.47, AI Cognitive Application *M* = 3.42). They possess basic AI ethical cognition and application ability, with moderate motivation and behavioral engagement. This group represents the core potential for AI literacy improvement.
3. High ethics-low self-efficacy type (19.4%): This group is characterized by outstanding AI ethical awareness (*M* = 4.31, the highest among all groups) but notably lower self-efficacy (*M* = 2.87, the lowest among all groups) and cognitive application (*M* = 3.12). These pre-service teachers demonstrate strong recognition of AI ethical issues such as fairness and privacy protection, but lack confidence in their ability to master AI knowledge and apply AI tools effectively. This profile reveals a disconnection between cognitive recognition and ability beliefs, which aligns with Expectancy-Value Theory’s proposition that ability beliefs are a necessary prerequisite for behavioral engagement and ability development.
4. Overall low-level type (11.5%): This group shows low scores across all dimensions (all dimension scores <3.0, with AI Cognitive Application *M* = 2.63 and AI Behavioral Commitment *M* = 2.71). Their AI ethical cognition, motivation, ability beliefs, and behavioral engagement are all at low levels, indicating insufficient understanding and willingness to apply AI technology. This group requires focused attention and intervention for AI literacy development.

**Table 8:** Exploratory profile analysis of AI literacy.

Profile type	Proportion	Five-factor average score (1–5)	Core characteristic description
Overall high-level type	27.80%	Ethics = 4.23, self-efficacy = 4.15, intrinsic motivation = 4.08, behavioral commitment = 4.02, cognitive application = 3.97	All dimensions develop balanced and at high levels, with strong AI ethical awareness, sufficient self-efficacy and intrinsic motivation, serving as the benchmark group for AI literacy cultivation.
Medium-level type	41.30%	Ethics = 3.65, self-efficacy = 3.58, intrinsic motivation = 3.52, behavioral commitment = 3.47, cognitive application = 3.42	All dimensions develop relatively balanced but at medium levels, with basic AI ethical cognition and application ability, representing the core potential group for AI literacy improvement.
High ethics-low self-efficacy type	19.40%	Ethics = 4.31 (highest), self-efficacy = 2.87 (lowest), other dimensions = 3.12–3.25	Outstanding AI ethical awareness, but lacking confidence in mastering AI knowledge and applying AI tools, showing characteristics of disconnection between cognitive recognition and ability beliefs.
Overall low-level type	11.50%	All dimension scores <3.0, with cognitive application = 2.63, behavioral commitment = 2.71	AI ethical cognition, motivation, ability beliefs and behavioral engagement are all at low levels, representing the key group requiring AI literacy intervention.

The four profiles showed significant differences in AI teaching integration ability (*F* = 42.87, *p* < 0.001), with Overall High-level Type students scoring significantly higher (*M* = 4.12) than other groups, while Overall Low-level Type students scored the lowest (*M* = 2.85). This indicates that the five-factor AI literacy structure effectively reflects pre-service teachers’ actual AI literacy levels and is highly correlated with their core professional competency—AI teaching integration ability. For educational evaluation, the four profiles can be directly used for group identification and differentiated feedback in formative assessment. For example, evaluators can assign different assessment tasks to each profile: performance-based tasks for the High Ethics-Low Self-efficacy group, and motivation-activation activities before skill testing for the Overall Low-level group.

Furthermore, grade development characteristics were observed in the profiles. Among second-year students, the proportion of High Ethics-Low Self-efficacy type reached 31.4%, while among third-year students this proportion dropped to 24.2%. Conversely, the proportion of Overall High-level type among third-year students (22.7%) was higher than among second-year students (16.0%), and third-year students’ average score on AI Self-efficacy dimension was significantly higher than second-year students (*p* = 0.035). This pattern suggests that as pre-service teachers progress through their university studies, accumulating more AI-related coursework, teaching practice preparation, and hands-on project experience, their confidence in AI abilities gradually improves, and the imbalance between ethical cognition and ability beliefs diminishes. However, except for AI Intrinsic Motivation, other dimensions did not show significant grade differences, indicating that the overall development pattern of pre-service teachers’ AI literacy requires further verification with larger longitudinal samples.

## 4 Discussion

### 4.1 Main psychometric findings

This study translated and adapted the AI Literacy Questionnaire for use with Chinese pre-service teachers. The five-factor structure (AI Ethics, AI Behavioral Commitment, AI Self-efficacy, AI Cognitive Application, AI Intrinsic Motivation) was retained as the preferred model based on both statistical and theoretical considerations. The model demonstrated acceptable fit using WLSMV estimation, and cross-method robustness was supported by consistent findings with ML/MLR estimation.

Internal consistency reliability was acceptable to strong across all factors (Cronbach’s *α* = 0.80–0.90), and initial evidence supported convergent validity (AVE > 0.50, CR > 0.84) and discriminant validity (HTMT < 0.85). The separation of the original affective dimension into distinct AI Self-efficacy and AI Intrinsic Motivation factors, as hypothesized through Expectancy-Value Theory, is further supported by their discriminant validity (HTMT = 0.40) and differential correlations with AI teaching integration ability. This structural differentiation is theoretically meaningful: self-efficacy (ability beliefs) and intrinsic motivation (task value) represent distinct but related motivational pathways that EVT posits contribute differently to achievement outcomes. Measurement invariance testing across gender groups supported configural invariance, establishing that the same five-factor structure is conceptually meaningful for both male and female participants. However, metric and scalar invariance were not established, which precludes direct comparison of latent means or observed total scores across gender. As detailed in Section 3.5, this limitation likely reflects the limited male sample size (*n* = 75) and potential genuine differences in item interpretation across gender groups, and should be addressed in future research with more balanced samples.

Given the absence of metric invariance, comparisons of latent means or observed total scores between male and female pre-service teachers are not recommended. Instead, researchers may compare gender differences at the item level or use alignment optimization to approximate invariance. For applied evaluation, we suggest reporting gender-specific norms separately rather than pooling scores.

### 4.2 Structural interpretation

The five-factor structure identified in this study differs from the original four-factor ABCE framework in two notable ways, both of which were theoretically anticipated through our pre-specified Expectancy-Value Theory framework. First, the affective learning dimension split into two separate factors (AI Self-efficacy and AI Intrinsic Motivation), confirming our hypothesis derived from EVT that ability beliefs and task value would emerge as distinct constructs in this population. As pre-service teachers’ AI learning is oriented toward future professional application rather than general interest, the psychological distinction between confidence in one’s capabilities (self-efficacy as ability beliefs) and perceived importance of AI learning (intrinsic motivation as task value) becomes more salient. This finding aligns with EVT’s core proposition that these motivational components, while correlated, contribute independently to learning outcomes. The differential predictive patterns observed in criterion-related validity testing—where AI Ethics, Behavioral Commitment, and Cognitive Application significantly predicted AI teaching integration while self-efficacy and intrinsic motivation showed non-significant or marginal effects—further support the theoretical value of maintaining these constructs as separate dimensions for assessment purposes. These patterns may reflect the early stage of AI education among pre-service teachers, where domain-specific competencies (ethics, commitment, cognition) may be more proximal predictors of teaching integration than general motivational beliefs, which may become more influential as teachers gain classroom experience.

Second, the behavioral learning dimension was integrated into a single AI Behavioral Commitment factor, with collaboration items failing to form an independent factor. One possible explanation is that pre-service teachers’ collaborative behaviors are often embedded within specific learning task commitments, making them difficult to distinguish as independent behavioral characteristics. The ethical learning dimension, in contrast, maintained its structural stability across both versions, with all eight items loading on a single AI Ethics factor. This pattern may indicate that ethical considerations were more consistently organized in the present sample (fairness, privacy, responsibility), though formal cross-cultural equivalence testing would be needed to confirm this interpretation. These structural differences should be interpreted cautiously, as they may reflect the combined influence of educational stage, group identity, and cultural context rather than any single factor.

### 4.3 AI literacy profiles and differentiated characteristics

Based on the five-factor scores of the Chinese version of the scale, this study identified the heterogeneity of pre-service teachers’ AI literacy through K-means cluster analysis. The optimal number of clusters was determined to be four based on three statistical indicators: silhouette coefficient, Calinski-Harabasz index, and elbow method. The classification results not only demonstrate the group heterogeneity of pre-service teachers’ AI literacy but also provide clear diagnostic evidence for precise and differentiated AI literacy cultivation, while further validating the scale’s discriminant validity—the ability to effectively identify different characteristic groups meets the practical needs of localized assessment.

From the perspective of profile formation mechanisms, grade differences are important background variables reflecting literacy development characteristics, mainly manifested in the proportion changes of the High Ethics-Low Self-efficacy type and the improvement of ability belief dimensions. Group analysis shows that among second-year students, the proportion of High Ethics-Low Self-efficacy type reached 31.4%, while among third-year students this proportion dropped to 24.2%. At the same time, the proportion of Overall High-level type among third-year students (22.7%) was higher than among second-year students (16.0%), and third-year students’ average score on AI Self-efficacy dimension was significantly higher than second-year students (*p* = 0.035). This difference reflects that as pre-service teachers progress through their university studies, accumulating more AI-related coursework, teaching practice preparation, and hands-on project experience, their confidence in AI abilities gradually improves, and the imbalance between ethical cognition and ability beliefs is significantly ameliorated, showing a gradual and progressive development characteristic in the ability belief dimension.

Interpreted through Expectancy-Value Theory, the four profiles reveal distinct motivational configurations with clear implications for instruction. Overall High-level students possess both high ability beliefs and high task value, consistent with EVT’s proposition that dual high motivational beliefs drive positive learning outcomes. These students demonstrate the highest AI teaching integration ability and serve as benchmarks for peer learning. However, the non-significant prediction of teaching integration by self-efficacy and intrinsic motivation in the SEM analysis suggests that for pre-service teachers at this developmental stage, domain-specific competencies (ethics, commitment, cognition) may be more proximal predictors of teaching readiness than general motivational beliefs. This pattern does not disconfirm EVT but rather identifies a boundary condition: motivational beliefs may become stronger predictors as teachers accumulate classroom experience. The High Ethics-Low Self-efficacy group exemplifies EVT’s proposition that high task value without corresponding ability beliefs limits behavioral engagement; their outstanding ethical awareness does not translate into strong cognitive application due to low confidence. The Overall Low-level group presents the expected dual-deficiency pattern, while the Medium-level group represents the largest intervention opportunity with moderate levels across both motivational constructs.

These profile characteristics, interpreted through EVT, provide a framework for differentiated instructional design. For the High Ethics-Low Self-efficacy group (19.4%), who possess high task value but low ability beliefs, interventions should leverage their ethical cognition strengths through case-based learning while building self-efficacy through scaffolded, step-by-step AI tool training with phased feedback. For the Overall Low-level group (11.5%), exhibiting dual deficiency in both motivational constructs, a motivation-activation approach should precede skill training—showcasing AI applications in teaching contexts to establish perceived utility before introducing basic AI competencies. For the Medium-level group (41.3%), peer-assisted learning with High-level students can drive engagement while targeted strengthening of weaker dimensions supports steady improvement. For the Overall High-level group (27.8%), advanced activities such as AI-integrated lesson design and peer mentoring deepen their capabilities while harnessing their benchmark potential. These differentiated approaches transform homogeneous instruction into targeted empowerment based on each profile’s specific motivational configuration.

### 4.4 Implications for educational evaluation

The validated AILQ has practical implications for educational evaluation. First, the five-factor structure enables pre-post program evaluation to assess AI curriculum effectiveness. Second, the four learner profiles provide diagnostic targets for formative assessment: for example, the High Ethics-Low Self-efficacy group requires performance-based evaluation rather than additional ethics training, while the Overall Low-level group needs basic motivation activation. Third, the scale can be used for system-level monitoring across institutions to identify regional or demographic gaps in AI literacy. The significant associations of AI Ethics, Behavioral Commitment, and Cognitive Application with AI teaching integration ability (*β* = 0.13–0.29) support using these dimensions as predictive indicators of teaching readiness. Overall, the AILQ serves as a flexible evaluation tool for both formative and summative purposes in teacher education. Given that the sample was drawn solely from Guangdong Province, institutions in other regions should first establish cross-regional measurement invariance before applying the norms or profile classifications derived here.

### 4.5 Limitations

This study has several limitations that should be considered when interpreting the findings. First, the sample was drawn exclusively from universities in Guangdong Province, limiting external generalizability to other regions of China with different educational resources and policy implementation contexts. Second, the sample composition was heavily imbalanced: 86.5% of participants were third-year students and 78% were female. While the female predominance reflects the broader demographic reality of Chinese teacher education programs (where female enrollment typically exceeds 70%), this imbalance limits generalizability to male-dominated teaching contexts and other countries with different gender distributions. The grade concentration similarly constrains conclusions about AI literacy development across the full teacher preparation trajectory; grade-related findings should be considered hypothesis-generating rather than confirmatory given the small first-year (*n* = 2) and fourth-year (*n* = 9) subsamples. Third, the study relied on self-report data and employed a cross-sectional design, which precludes causal inferences and may be subject to social desirability bias. Fourth, the EFA was conducted using Pearson correlation matrices rather than polychoric correlations, which may be less optimal for ordinal categorical data; however, this was partially addressed through WLSMV confirmatory factor analysis. Fifth, formal cross-cultural measurement invariance testing was not conducted; within-sample gender invariance testing supported only configural invariance, with metric and scalar invariance not established, likely due in part to the limited male sample size (*n* = 75).

Finally, because the dataset did not include class or school identifiers, we could not account for potential clustering effects (e.g., students nested within classes or schools). Ignoring such clustering may lead to underestimated standard errors and inflated Type I error rates (McNeish et al., 2017). Although the present sample was drawn from multiple universities and classes, the absence of nested identifiers precluded multilevel modeling or the calculation of intraclass correlation coefficients (ICCs). Future research should collect hierarchical data and apply multilevel confirmatory factor analysis (MCFA) or design-based corrections to examine whether the factor structure and parameter estimates are robust to clustering. In the meantime, our single-level results should be interpreted as providing initial evidence pending replication with clustered data.

## 5 Conclusion

This study provides initial psychometric evidence for the Chinese version of the AI Literacy Questionnaire among pre-service teachers in Guangdong Province. Given that the sample was drawn exclusively from this province and predominantly from third-year students, the findings should be considered specific to this population pending cross-regional and cross-grade validation. The five-factor structure (AI Ethics, AI Behavioral Commitment, AI Self-efficacy, AI Cognitive Application, AI Intrinsic Motivation) was retained as the preferred model, demonstrating acceptable fit and cross-method robustness.

The exploratory profile analysis identified four distinct AI literacy profiles among pre-service teachers: Overall High-level Type (27.8%), Medium-level Type (41.3%), High Ethics-Low Self-efficacy Type (19.4%), and Overall Low-level Type (11.5%). These profiles exhibit significant grade development characteristics, with third-year students showing improved self-efficacy and reduced proportion of the High Ethics-Low Self-efficacy type compared to second-year students, suggesting that accumulated learning experiences contribute to more balanced AI literacy development.

The profile analysis offers preliminary practical insights for differentiated instructional approaches in teacher education. The distinct motivational and ability patterns identified across profiles suggest that targeted interventions may be more effective than one-size-fits-all approaches. For instance, the High Ethics-Low Self-efficacy group may benefit from confidence-building activities that bridge their strong ethical awareness with practical skill development, while the Overall Low-level group may require foundational motivation activation before skill training.

However, further validation with more diverse samples is needed before these classifications can be used for systematic intervention design. Future research should expand the sample to include multiple provinces and grade levels, employ longitudinal designs to track profile development over time, and evaluate the effectiveness of differentiated interventions tailored to specific profile characteristics. The Chinese version of the AI Literacy Questionnaire provides a psychometrically sound foundation for such future research and for practical application in teacher education programs seeking to prepare future educators for an AI-enhanced teaching landscape.

## Data Availability

The original contributions presented in the study are included in the article/Supplementary material, further inquiries can be directed to the corresponding author.
