# Supplementary material for: Validation of the AI literacy questionnaire for Chinese pre-service teachers: psychometric evidence and profiles for differentiated educational evaluation
Source: Front Psychol. 2026 Jun 22;17:1854432. doi: 10.3389/fpsyg.2026.1854432 (PMC13333676; doi:10.3389/fpsyg.2026.1854432)
Supplement: Supplementary file 1 [file Supplementary_file_1.DOCX]

**Supplementary Material B**

Theoretical Justification for EFA Item Deletion

In the exploratory factor analysis (EFA), eight items were deleted from the original 32-item pool, resulting in a final 24-item, five-factor structure. While statistical criteria (factor loadings, cross-loadings, and communalities) guided the initial screening, theoretical justification is essential for transparent and interpretable scale refinement. Drawing on Expectancy-Value Theory (Eccles & Wigfield, 2002) and the professional characteristics of pre-service teachers, the following sections provide detailed theoretical rationale for the deletion of each item.

# 1. Collaboration and Behavioral Commitment Items (4 items deleted)

Four items from the original behavioral learning dimension that specifically addressed collaborative AI learning failed to load independently. These items are: BA1, BB1, BB2, and BB3.

| **Item** | **Original Item Content (English)** |
| --- | --- |
| BB1 | I often try to explain the AI learning materials to my classmates or friends. |
| BB2 | I try to work with my classmates to complete AI learning tasks and projects. |
| BB3 | I often spend spare time discussing AI with my classmates. |
| BA1 | I will continue to use AI in the future. |

Theoretical rationale: Theoretically, this deletion pattern is consistent with the professional orientation of pre-service teachers, whose collaborative behaviors may be embedded within specific task commitments rather than manifesting as independent behavioral characteristics (Younas et al., 2025c). For this population, behavioral commitment to AI learning is primarily expressed through individual professional preparation rather than peer collaboration.

Specifically, BB1-BB3 target peer-oriented collaborative behaviors (explaining materials to classmates, working with classmates on tasks, discussing AI with classmates during spare time). For pre-service teachers in mainland China, such collaborative behaviors are typically situated within structured course assignments or teaching practicum tasks rather than emerging as independent behavioral tendencies. As a result, these items did not form a distinct factor in the EFA.

BA1 ("I will continue to use AI in the future") focuses on a general behavioral intention toward AI technology use, which differs from the active learning commitment captured by the retained items (BA2-BA5). BA1's phrasing connotes passive technology adoption rather than proactive professional engagement with AI, making it conceptually less aligned with the behavioral commitment factor as operationalized for pre-service teachers.

# 2. Affective Cross-loading Items (2 items deleted)

Two items that originally measured general affective responses cross-loaded between the self-efficacy and intrinsic motivation factors. These items are: AC1 and AC3.

| **Item** | **Original Item Content (English)** |
| --- | --- |
| AC1 | I can understand AI related resources/tools. |
| AC3 | I feel confident that I will do well in the AI related tasks. |

Theoretical rationale: This cross-loading pattern is theoretically consistent with EVT's proposition that ability beliefs and task value, while related, are distinct constructs (Eccles & Wigfield, 2002; Afzaal et al., 2025). The cross-loading suggests these items were ambiguously worded for a population where professional application (task value) and confidence (ability beliefs) are closely intertwined.

Item AC1 ("I can understand AI related resources/tools") simultaneously invokes a capability judgment ("can understand"; ability belief) and an engagement with AI resources (task value). Similarly, AC3 ("I feel confident that I will do well") combines an affective state ("feel confident") with an outcome expectation ("do well"), rendering it difficult for pre-service teachers to disentangle their confidence in AI abilities from their interest in AI learning.

For pre-service teachers, whose AI learning is oriented toward future professional application, ability beliefs and task value are more psychologically intertwined than among general student populations. The deletion of AC1 and AC3 enhances the construct purity of the self-efficacy and intrinsic motivation factors, ensuring that each factor captures a distinct motivational pathway as postulated by EVT.

# 3. Cognitive Knowledge Items (2 items deleted)

Two items assessing basic AI knowledge awareness were deleted due to low communalities. These items are: CA2 and CB1.

| **Item** | **Original Item Content (English)** |
| --- | --- |
| CA2 | I know how to use AI applications (e.g., Siri, chatbot). |
| CB1 | I can apply AI applications to solve problems. |

Theoretical rationale: Theoretically, this reflects the ceiling effect among pre-service teachers in Guangdong Province, where basic AI concepts are already integrated into the curriculum (Younas et al., 2025a). The retained cognitive application items better capture the practical knowledge dimension relevant to teaching practice.

Item CA2 assesses declarative knowledge of common AI applications (e.g., virtual assistants, chatbots), while CB1 targets basic application skills ("apply AI applications to solve problems"). Both items measure foundational AI competencies that have been increasingly incorporated into the teacher education curriculum in Guangdong Province under national initiatives such as the Education Informatization 2.0 Action Plan. Consequently, the majority of pre-service teachers in the sample already possessed these basic competencies, resulting in restricted variance and low communalities.

The retained cognitive items (CA1: AI definition; CA5: comparing AI concepts; CB3: creating AI-driven solutions; CB4: evaluating AI applications) assess higher-order cognitive processes that are more differentiating among pre-service teachers and more directly relevant to their future pedagogical practice. These items capture the ability to analyze, evaluate, and create with AI—skills that are essential for effective AI integration in teaching but less uniformly distributed in the sample.

# 4. Summary of Deleted Items

| **Category** | **Items Deleted** | **Core Theoretical Rationale** |
| --- | --- | --- |
| Collaboration and Behavioral Commitment | 4 (BB1-BB3, BA1) | Collaborative behaviors embedded within task commitments; not independent behavioral characteristics. |
| Affective Cross-loading | 2 (AC1, AC3) | Ability beliefs and task value are distinct constructs (EVT); items ambiguously worded for this population. |
| Cognitive Knowledge | 2 (CA2, CB1) | Ceiling effect due to basic AI concepts already integrated into the curriculum. |

In conclusion, the deletion of these eight items is supported by both statistical evidence and theoretical reasoning grounded in Expectancy-Value Theory and the professional characteristics of pre-service teachers. The resulting 24-item, five-factor structure (AI Ethics, AI Behavioral Commitment, AI Self-efficacy, AI Cognitive Application, AI Intrinsic Motivation) provides a psychometrically sound and theoretically coherent instrument for assessing AI literacy among mainland Chinese pre-service teachers.
