# Supplementary material for: Validation of the AI literacy questionnaire for Chinese pre-service teachers: psychometric evidence and profiles for differentiated educational evaluation
Source: Front Psychol. 2026 Jun 22;17:1854432. doi: 10.3389/fpsyg.2026.1854432 (PMC13333676; doi:10.3389/fpsyg.2026.1854432)
Supplement: Supplementary file 2 [file Supplementary_file_2.DOCX]

**Supplementary Material**

**A. Detailed data cleaning results**

This study used multi-indicator joint screening methods to identify careless responses. Specific screening results are:

1. Longstring analysis: Samples with Longstring ≥ 15 were 137 (34.5%), with Longstring = 32 (all answers same) being 20 (5.0%).

2. Response variability analysis: Samples with standard deviation < 0.3 were 38 (9.6%).

3. Response range analysis: Samples with range = 0 were 20 (5.0%), samples with range ≤ 1 were 76 (19.1%).

4. Comprehensive screening: Samples meeting at least 2 screening criteria were 56 (14.1%), which were deleted.

**B. Full EFA loading matrix**

Factor loading matrix using MinRes extraction combined with Promax oblique rotation (|loading| ≥ 0.40):

Factor 1 (AI Ethics, 30.10% variance): DA12(0.855), DA10(0.833), DA5(0.821), DA11(0.763), DA7(0.720), DA13(0.701), DA8(0.677), DA2(0.646)

Factor 2 (AI Behavioral Commitment, 12.69% variance): BA3(0.860), BA2(0.812), BA5(0.780), BA4(0.753)

Factor 3 (AI Self-efficacy, 6.44% variance): AB2(0.870), AB4(0.823), AB1(0.777), AB3(0.736)

Factor 4 (AI Cognitive Application, 5.87% variance): CA5(0.852), CB4(0.793), CB3(0.698), CA1(0.677)

Factor 5 (AI Intrinsic Motivation, 4.19% variance): AA3(0.899), AA1(0.806), AA2(0.801), AA4(0.567)

Note: This study's EFA is exploratory analysis phase results, containing 32 initial items; subsequent deletion of 8 unqualified items for CFA confirmatory analysis (24 items).

**C. Full CFA standardized loadings**

Standardized factor loadings from confirmatory factor analysis (WLSMV/DWLS estimation):

AI Ethics: DA2(0.599), DA5(0.751), DA7(0.739), DA8(0.654), DA10(0.788), DA11(0.746), DA12(0.787), DA13(0.745)

AI Behavioral Commitment: BA2(0.723), BA3(0.776), BA4(0.795), BA5(0.750)

AI Self-efficacy: AB1(0.780), AB2(0.805), AB3(0.719), AB4(0.789)

AI Cognitive Application: CA1(0.643), CA5(0.713), CB3(0.695), CB4(0.797)

AI Intrinsic Motivation: AA1(0.709), AA2(0.778), AA3(0.778), AA4(0.716)

WLSMV vs ML comparison: Differences between WLSMV and ML estimates were minimal (ΔCFI = 0.01, ΔRMSEA = 0.015), supporting the stability of the five-factor structure across estimation methods.

**D. Additional fit indices and cluster diagnostics**

WLSMV/DWLS confirmatory factor analysis fit indices:

χ² = 166.10, df = 242, χ²/df = 0.69

CFI = 0.960, TLI = 0.953, RMSEA = 0.035

GFI = 0.970, AGFI = 0.966, NFI = 0.970

SRMR = 0.069, AIC = 112.85, BIC = 335.10

Cluster analysis diagnostics:

Silhouette Coefficient = 0.72 (indicating good clustering effect)

Calinski-Harabasz Index = 186.3 (peak value)

Elbow method: SSE decline rate significantly slowed after 4 clusters

Cross-validation results:

First half sample CFI = 0.939

Second half sample CFI = 0.909

Full sample CFI = 0.945 (WLSMV) / 0.945 (ML)

Grade difference analysis for exploratory profile interpretation:

Among second-year students, the proportion of High Ethics-Low Self-efficacy type reached 31.4%, while among third-year students this type proportion dropped to 24.2%. The proportion of Overall High-level type among third-year students (22.7%) was higher than among second-year students (16.0%), and third-year students' average score on AI Self-efficacy dimension was significantly higher than second-year students (p = 0.035).
